# Complete mitochondrial genome and phylogenetic analysis of *Schizothorax sinensis* (Teleostei: Cypriniformes: Cyprinidae)

**DOI:** 10.1080/23802359.2021.1901622

**Published:** 2021-03-19

**Authors:** Deng Chen, Changkun Fu, Ziyong Lei, Yuhao Li, Tianmeng Zhao, Hailing Fan, Tao Hu, Qian Wang, Hao Zong

**Affiliations:** College of Life Sciences, Sichuan Normal University, Chengdu, China

**Keywords:** Mitochondrial genome, *Schizothorax sinensis*, phylogenetic tree, Schizothoracinae

## Abstract

This study describes the first sequencing of the complete mitochondrial genome of *Schizothorax sinensis*, a species of cyprinid snowtrout from the Jialing River and Fujiang River basins in China’s Sichuan Province. The total length is 16,571 base pairs. Similar to most Schizothoracinae mitochondrial genomes, there are 37 genes including 13 protein coding genes, 22 transfer RNA genes and 2 ribosomal RNA genes. In addition, it contains a control region rich in A-T nucleotides. The overall nucleotide composition is 29.6% for A, 27.1% for C, 17.9% for G and 25.4% for T, and the percentage of GC content is 45.0%. Phylogenetic analysis suggested that *Schizothorax sinensis* and *Schizothorax prenanti* clustered together in a clade. This work provides additional molecular information for studying *Schizothorax sinensis* conservation genetics and evolutionary relationships.

*Schizothorax sinensis* (Herzenstein, 1889) is endemic to the middle and upper reaches of the Yangtze River, which allies within order Cypriniformes (subfamily Schizothoracinae; family Cyprinidae) (Ding 1994). This species is frequently found in clustered groupings in cold water, high-flow reaches of streams and rivers in the upper reaches of the Jialing River canyon in the Sichuan Province of China, including the Fujiang River and Qujiang River (Li et al. 2008), where it is important as a fishery (Leng et al. 1984). We analyzed the sequence information and structural characteristics of the complete mitochondrial genome of *Schizothorax sinensis*, and conducted a comparative phylogenetic analysis relative to existing mitogenomes of other schizothoracinae (snowtrout and allies) fishes of Family Cyprinidae from rivers and lakes in central Asia. .

Specimen of *Schizothorax sinensis* was collected from Jiaojiahe section of the Jialing River, Bazhong City, Sichuan Province, China, on 3 July 2020 (Latitude: 32°39′19.62″N, Longitude: 106°40′52.34″E, Altitude: 947 m), and is currently stored in the Zoological Museum, College of Life Sciences, Sichuan Normal University, China (Specimen number: BZ-20-07002, 95% alcohol). In this study, the complete mitochondrial genome sequence was obtained by high-throughput sequencing with Illumina Hiseq 2500 (Tsingke, Beijing) and then assembled with SPAdes v3.13.0 (Bankevich 2012) and annotated with GeSeq (Tillich 2017). The complete mitogenome sequence of *Schizothorax sinensis* was deposited into GenBank database with the accession number MW191514.

The complete mitochondrial genome of *Schizothorax sinensis* is similar to other Schizothoracinae species (Zhang et al. 2018, Chen et al. 2016), which is 16,571 bp in length and reads 13 protein-coding genes (PCGs: ND1, ND2, ND3, ND4, ND4L, ND5, ND6, COI, COII, COIII, ATP6, ATP8 and Cytb), 22 Transfer RNA genes (tRNA), 2 ribosomal RNA genes (rRNA) and 2 non-coding regions: A-T rich control region (D-loop) and origin of light-strand replication (OL). The nucleotide composition is 29.6% for A, 27.1% for C, 17.9% for G and 25.4% for T. The large ribosomal RNA (lrRNA) is 1631 bp in length and small ribosomal RNA (srRNA) is 954 bp in length. The length of the control region is 805 bp.

Based on the concatenated nucleotide sequence of the complete mitogenome, the phylogenetic relationships of the *Schizothorax sinensis* and the other 16 fishes were constructed by MEGA6.0 using maximum-likelihood (ML) method with 1000 bootstrap replications (Tamura et al. 2013). The phylogenetic tree (Figure 1) showed that the *Schizothorax sinensis* was closer to *Schizothorax prenanti* in genetic relationship (Chen et al. 2016), which is consistent with the results of traditional morphological classification (Ding 1994). That is, our mitogenome phylogenetic analysis substantiates *Schizothorax sinensis* belonging to genus *Schizothorax* of the subfamily Schizothoracinae. The tree supports clear phylogenetic relationships at the genus level. This complete mitogenome provides a foundation for future studies investigating the phylogenetic relationships of genus *Schizothorax* and other schizothoracine fishes, including studies into their biogeography, conservation genetics and evolutionary relationships.

**Figure 1.**
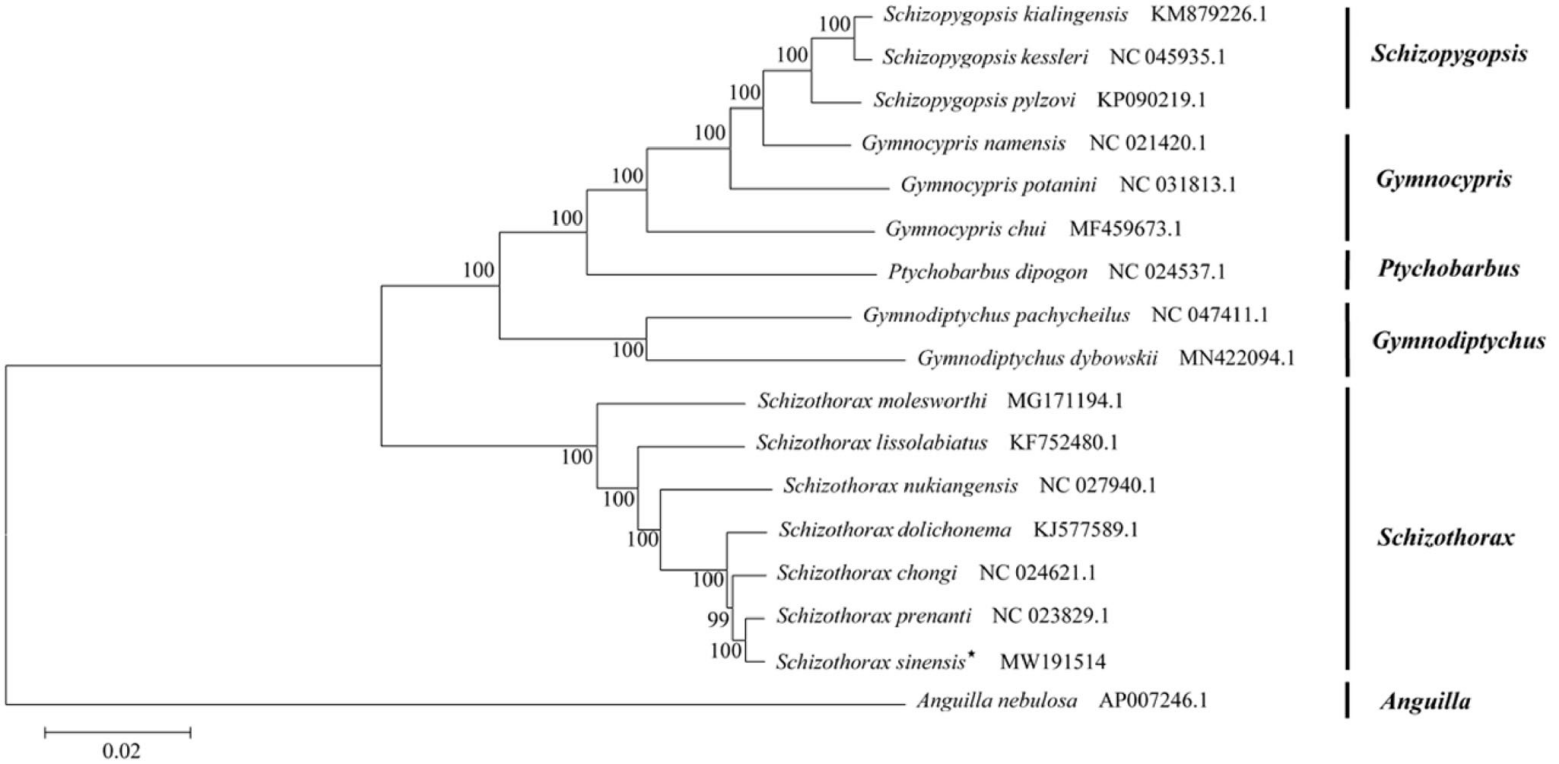
Phylogenetic tree inferred from Maximum Likelihood analysis of the nucleotide of complete mitochondrial genome. *Anguilla nebulosa* was used as outgroups. The nodal numbers indicate the bootstrap values obtained with 1000 replicates. The species name, GenBank accession number and genus name were shown on the right side of the phylogenetic tree. The newly sequenced mitogenome is indicated by the asterisk.

## Data Availability

The genome sequence data that support the findings of this study are openly available in GenBank of NCBI at (https://www.ncbi.nlm.nih.gov/) under the accession no. MW191514. The associated BioProject, SRA, and Bio-Sample numbers are PRJNA702895, SRR13749644, and SAMN17982894, respectively.

## References

[CIT0001] Bankevich A, Nurk S, Antipov D, Gurevich AA, Dvorkin M, Kulikov AS, Lesin VM, Nikolenko SI, Pham S, Prjibelski AD, et al. 2012. SPAdes: a new genome assembly algorithm and its applications to single-cell sequencing. J Comput Biol. 19(5):455–477. 22506599 10.1089/cmb.2012.0021 PMC3342519

[CIT0002] Chen CN, Huang YY, Li H, Long ZH, Lai JS, Liu GX, Zhao G. 2016. The complete mitochondrial genome of *Schizothorax prenanti* (Tchang) (Teleostei, Cyprinidae, Schizothoracinae). Mitochondrial DNA A DNA Mapp Seq Anal. 27(1):253–254. 24521499 10.3109/19401736.2014.883612

[CIT0003] Ding RH. 1994. The fishes of Sichuan, China. Chengdu: Sichuan Publishing House of Science and Technology. p. 368–370. (in Chinese)

[CIT0004] Leng YZ, Zhou ZQ, Huang DX. 1984. Biological data of *Schizothorax sinensis*. Chin J Zool. 19(6):45–47. (in Chinese)

[CIT0005] Li H, Du J, Wu MS, Guo J. 2008. Status quo and protection measures of *Schizothorax sinensis*. J Hydroecol. 28(2):110–111. (in Chinese)

[CIT0006] Tamura K, Stecher G, Peterson D, Filipski A, Kumar S. 2013. MEGA6: Molecular Evolutionary Genetics Analysis version 6.0. Mol Biol Evol. 30(12):2725–2729. 24132122 10.1093/molbev/mst197 PMC3840312

[CIT0007] Tillich M, Lehwark P, Pellizzer T, Ulbricht-Jones ES, Fischer A, Bock R, Greiner S. 2017. GeSeq–versatile and accurate annotation of organelle genomes. Nucl Ac Res. 45(W1):W6–W11. 10.1093/nar/gkx391 PMC5570176 28486635

[CIT0008] Zhang C, Ma B, Mey G, Liu HP, Wang WL, Li BH, Zhang BB. 2018. Characterization of the complete mitochondrial genome of the endangered species *Schizothorax integrilabiatus*. Mitochondrial DNA B Resour. 3(1):309–310. 33474155 10.1080/23802359.2018.1445486 PMC7799548

